# Factors Associated with Up-to-Date Measles Vaccination Among 2-Year-Old Children in Canada: An Analysis of the 2021 Childhood National Immunization Coverage Survey

**DOI:** 10.3390/vaccines14090817

**Published:** 2026-09-17

**Authors:** Jeanette Bourne, Anna-Maria Frescura, Samuel Ileka-Priouzeau, Marwa Ebrahim, Julie Laroche

**Affiliations:** Vaccine Coverage and Effectiveness Surveillance Division, Infectious Diseases and Vaccination Programs Branch, Public Health Agency of Canada, Ottawa, ON K1A 0K9, Canada; jeanette.bourne@phac-aspc.gc.ca (J.B.);

**Keywords:** measles vaccine, immunization, children, Canada

## Abstract

**Background/Objectives**: Ongoing measles outbreaks in Canada and worldwide, largely among unvaccinated individuals, emphasize the importance of examining predictors of measles vaccination. This study aims to report on factors associated with up-to-date measles vaccination among 2-year-olds in Canada. **Methods**: We used national cross-sectional data from the 2021 childhood National Immunization Coverage Survey (cNICS), which was conducted among 2-year-old Canadian children between 10 January 2022 and 14 July 2022. We examined proportions of vaccinated children by sociodemographic characteristics and conducted multivariable logistic regression analysis to determine factors associated with up-to-date measles vaccination coverage, defined as receiving at least one dose of a measles-containing vaccine by 2 years of age. **Results**: Among 3118 two-year-olds, 91.6% (95% CI: 89.9–93.1) were up-to-date with their measles vaccines. Lower odds of being up-to-date among 2-year-olds was associated with a household income of less than $60,000 (aOR = 0.35, 95% CI: 0.16–0.75; *p* < 0.05), experiencing pandemic-related obstacles or delays to vaccination (aOR = 0.23, 95% CI: 0.12–0.43, *p* < 0.05) and parents being more vaccine-hesitant (aOR = 0.49, 95% CI: 0.26–0.94, *p* < 0.05). **Conclusions**: Lower household income, experiencing pandemic-related obstacles, and parental hesitancy contribute to being not up-to-date on measles vaccination among children in Canada. These results support developing targeted programs and communication efforts to help increase measles vaccination coverage in Canada.

## 1. Introduction

Measles is a highly contagious vaccine-preventable disease caused by an airborne virus that can result in severe outcomes such as encephalitis and death [1]. There is no specific treatment for measles; however, vaccination is the most cost-effective way to prevent the disease. Despite global immunization efforts, the WHO reported that in 2024 approximately 95,000 deaths were attributed to measles, with the largest burden seen among unvaccinated or under-vaccinated children younger than 5 years of age [1]. Measles infection is also particularly dangerous among pregnant women as it can lead to preterm delivery and/or low birth weight [1]. Furthermore, measles infection may lead to “immune amnesia” which is loss of previously acquired immunity to other pathogens contributing to a long-term increase in susceptibility to other infections that can continue for years after apparent recovery from measles [2].

Low measles vaccination coverage in Canada and internationally is a barrier to achieving herd immunity and preventing measles outbreaks. Globally, in 2025, an estimated 29 million infants were not fully protected against measles [1]. Worldwide, 77% of children received two doses of the measles vaccine in 2025, unchanged from 2024 (77%) and slightly higher than in 2023 (74%), yet remaining well below the 2030 global target of 90%. In 2025, 84% of children worldwide received one dose of a measles vaccine by their second birthday which is on par with coverage measured in 2024 (84%) and 2023 (83%), but slightly lower than 2019 (86%) [2].

In Canada, adults born before 1970 are typically considered to have acquired natural immunity against measles; however, susceptible travellers to areas outside of Canada, healthcare workers, and military personnel should receive the measles vaccine, regardless of their year of birth. To ensure protection against measles, the National Advisory Committee on Immunization (NACI) recommends two doses of a measles-containing vaccine, with a first dose administered at 12 or 15 months of age and a second dose either at 18 months of age or any time thereafter but no later than school entry [3].

In Canada, routine childhood vaccines, including the measles vaccine, are publicly funded for children in all provinces and territories. Measles vaccination is offered as a combination vaccine either with mumps and rubella (MMR) or with mumps, rubella and varicella (MMRV) [4]. In 2021, 92% of children in Canada received one dose of a measles vaccine by their second birthday and 79% had received two doses by their seventh birthday; both of these estimates are below the 2025 national immunization coverage goal of 95% [5,6]. Vaccination coverage for measles is typically lower at 7 years of age compared to 2 yr olds because more doses are required to be up-to-date for measles vaccination for older age groups. In addition, younger children are more likely to be vaccinated for measles given that there are more healthcare encounters for vaccination at that age. While coverage has remained stable over time among 2-year-olds, measles vaccination coverage has decreased from 87% in 2017 to 79% in 2021 among seven-year-olds [5]. The Public Health Agency of Canada (PHAC) and its provincial and territorial partners established the Standardized Reporting on Vaccination (STARVAX) which reports on vaccination coverage among select provinces and territories using data provided from immunization registries or information systems. Across the five jurisdictions that reported on measles, mumps, rubella (MMR) vaccination coverage among 2-year-olds between 2019 and 2025 (Alberta, Manitoba, New Brunswick, Saskatchewan, and Yukon), coverage declined from 88.9% in 2019, to 84.6% in 2023, 80.2% in 2024, and 79.6% in 2025 [7].

Although Canada achieved measles elimination status in 1998 and maintained it for more than two decades, the country no longer held this status as of November 2025 due to ongoing multijurisdictional outbreaks that began in October 2024 [8]. In 2024, 146 measles cases and one measles death were reported [9]. The outbreaks intensified in 2025, with 5463 reported measles cases and two reported deaths among preterm infants caused by congenital measles [10]. As of 4 July 2026, 1097 measles cases had been reported in Canada, with the majority of cases reported among 5- to 17-year-olds (41%), unvaccinated individuals (86%), individuals residing in Manitoba (61%) and Alberta (28%) and those exposed in Canada, epidemiologically and/or virologically linked (96%) [11]. This resurgence reflects a broader global trend. Worldwide, measles cases have increased since 2019; there were 10.3 million cases in 2023, representing a 20% increase since 2022. As of April 2025, 61 countries experienced a large or disruptive measles outbreak in the preceding 12 months, which is the highest number of outbreaks since 2019 [12]. Understanding the factors associated with children being not up-to-date for measles vaccination is increasingly important for informing targeted public health interventions aimed at closing immunity gaps and preventing future outbreaks.

Under-vaccination or delays in childhood vaccination can increase children’s vulnerability to vaccine-preventable diseases such as measles. Previous studies in the United States have identified several sociodemographic characteristics associated with lower childhood vaccination coverage, including lower levels of maternal education and lower household income [13,14,15]. Children whose parents hold negative beliefs about vaccines are also more likely to be unvaccinated against measles [16]. In Canada, lower parental education is associated with total childhood non-vaccination, while measles non-vaccination is frequently reported among children of single-parent families and those of parents with lower education [17]. Additionally, barriers to accessing vaccination, such as lack of access to a healthcare provider, missed opportunities to vaccinate children and missed well-child visits, as well as parental vaccine hesitancy, have been identified as obstacles to on-time series completion and childhood vaccination coverage [13,16].

The PHAC monitors and reports on estimates of national vaccination coverage for all routine childhood vaccines, including the measles vaccine, through the Childhood National Immunization Coverage Survey (cNICS). Using data from the most recent cNICS cycle in 2021, our objectives were: (1) to estimate measles vaccination coverage and examine sociodemographic differences in vaccination status among 2-year-olds and (2) to identify factors associated with up-to-date measles vaccination among 2-year-olds in Canada, defined as receiving at least one dose of a measles-containing vaccine by two years of age [3]. The findings from this study will help improve our understanding of factors associated with Canadian 2-year-old children not being up-to-date for measles vaccination, which may help inform targeted public health strategies aimed at improving vaccination coverage and supporting Canada’s efforts to restore and sustain measles elimination.

## 2. Materials and Methods

### 2.1. Data Source

The current analysis uses data from the 2021 cNICS, a national cross-sectional survey, that measures the proportion of children in Canada who have received routine vaccinations by 2, 7, 14, and 17 years of age. The 2021 cNICS provides the most up-to-date national estimates on childhood vaccination coverage among children in Canada. Parents of 2- and 14-year-olds were also asked questions about their knowledge, attitudes and beliefs (KABs) towards vaccination. The cNICS has been implemented every two years by PHAC since 1994. Between 2013 and 2021, the survey was implemented by PHAC in collaboration with Statistics Canada. Data collection for the 2021 cNICS occurred from 10 January 2022 to 14 July 2022. The data collection phase of the cNICS involved the administration of a self-response electronic questionnaire or a computer-assisted telephone interview (CATI) during which the parent or guardian answered questions about the vaccines their child had received. The survey population is based on the list of applicants to the Canada Child Benefit (CCB), which is a monthly file provided by the Canada Revenue Agency. The difference between the target and survey populations relates to children of parents or guardians who did not apply for the CCB. This may include households that were unaware of the benefit or that decided not to apply for it. It may also include children who lived in foster care for the entire year, as their caregivers would have been subsidized by the provincial government. Children living on reserves and in other Indigenous communities in the provinces and the institutionalized population are also excluded from the survey. These exclusions represent less than 3% of the Canadian population.

The number of measles vaccine doses was determined by asking parents/guardians to review their child’s immunization booklet/card and report the date when each vaccine dose was received. If consent was provided, this information could also be verified using records from the child’s healthcare provider or their provincial immunization where data sharing agreements existed with Statistics Canada. In the 2021 cNICS, this was possible in Manitoba and Prince Edward Island only. Parental recall was not accepted as a valid response for reports on children’s immunization history.

The initial sample size was determined by setting quality targets on estimated proportions of the immunization coverage rate. The sample size was increased to account for non-response. A larger sample size is collected for 2-year-old children participating in the cNICS to allow for more detailed analysis.

### 2.2. Study Design

The 2021 cNICS sampling frame was designed using the Canadian Child Tax Benefit (CCB) list, which is stratified by age and province or territory and excludes children who live in foster care, those who reside on First Nations reserves, and those living in institutions (which constitutes less than 3% of Canadian children). Coverage estimates reported from the cNICS are weighted to adjust for non-response and post-stratification. The overall response rate for the 2021 cNICS was 43.9% (43.7% among parents/guardians of 2-year-olds). The detailed methodology of the cNICS has been previously published [18].

The 2021 cNICS was conducted by Statistics Canada under the Statistics Canada Act and was not considered health research; therefore, ethics board review and approval was not required.

### 2.3. Study Population

In the cNICS, measles vaccination includes vaccination by any measles-containing vaccine publicly available in Canada (measles, mumps, rubella (MMR) or measles, mumps, rubella, varicella (MMRV)) or formulations available outside Canada (monovalent measles vaccine). The study population for this analysis included 2-year-old-children with a known measles vaccination status, who resided in Canada at the time of data collection, and whose parents completed the 2021 cNICS questionnaire.

A total of 5446 parents/guardians in Canada participated in the 2021 cNICS, which included 3954 parents/guardians of 2-year-olds, 351 parents/guardians of 7-year-olds, 758 of 14-year-olds and 383 of 17-year-olds. The study sample included data on 3118 2-year-olds with known measles vaccination status.

### 2.4. Study Variables

#### 2.4.1. Dependent Variable

The dependent variable of interest was up-to-date measles vaccination status among 2-year-olds, defined as receiving at least one dose of a measles-containing vaccine by the child’s second birthday.

#### 2.4.2. Independent Variables

Independent predictor variables were selected from available indicators in the 2021 cNICS and prioritized based on the existing scientific literature [17,19].

##### Sociodemographic Characteristics

This study examined the following sociodemographic characteristics: region of residence (Atlantic, Ontario, Quebec, Prairies, British Columbia, and Territories), parent/guardian gender (man, woman), parent/guardian marital status (married/common, single), parent/guardian age (15–29, 30–39, 40–72 years), highest level of parent/guardian education (high school or less, above high school, below Bachelor’s (trades, colleges, certificate), Bachelor’s or higher), 2020 total household income (less than $60,000, $60,000–$99,999, $100,000–$149,999, 29.8, $150,000 and above), child’s gender (girl, boy), child’s ethnicity (Indigenous, White, Asian/Other, Black), and urban/rural. To address low sample sizes, category levels such as the region of residence variable were recoded. Newfoundland and Labrador, Prince Edward Island, Nova Scotia, and New Brunswick were combined as the “Atlantic Region,” and Manitoba, Saskatchewan and Alberta were grouped together as the “Prairies.” Data from the three territories were also grouped together as the “Territories”.

##### COVID-19 Pandemic-Related Obstacles or Delays to Vaccination

In the 2021 cNICS, respondents were asked if they experienced COVID-19 pandemic-related obstacles or delays to vaccinating their child. Parents were considered to have encountered a COVID-19 related delay or obstacles if they answered “yes” to either of the following two questions: ‘Excluding the COVID-19 and influenza vaccines, did you encounter any obstacles in getting [CHILD NAME] vaccinated due to the COVID-19 pandemic?’ or ‘Excluding the COVID-19 and influenza vaccines, have any vaccines for [CHILD NAME] been delayed by at least 30 days due to the COVID-19 pandemic?’

##### Vaccine Hesitancy Score

The vaccine hesitancy score is a continuous variable created by combining responses to six KAB statements about vaccine safety, vaccine effectiveness, stated importance of vaccination and trust in different sources of vaccine information. Appendix A—Table A1 includes the detailed statements used. This score is based on similar approaches used in other studies [20]. For each KAB statement, parents could respond if they “Strongly agree”, “Somewhat agree”, “Somewhat disagree”, or “Strongly disagree” with the statement. A composite score representing the average parental agreement towards these statements was created, such that strongly agree = 1, somewhat agree = 2, somewhat disagree = 3, and strongly disagree = 4. The vaccine hesitancy score was then created by deriving a mean score for the KAB statements, where the higher the score the higher the level of vaccine hesitancy based on more disagreement with the selected KAB statements.

### 2.5. Missing Values

For the outcome variable (measles vaccination status), only individuals with a known vaccination history were included; therefore, those indicating “don’t know” and “prefer not to answer” were removed from this analysis. For all predictor variables the responses “don’t know” and “prefer not to answer” were recoded as missing responses and excluded through listwise deletion. The total amount of missing data from these exclusions represents less than 5% of responses, which is unlikely to significantly affect overall results. Studies show that risk of bias is posed when 10% or more of cases have missing values [21,22].

### 2.6. Analysis

The characteristics of the study sample were first explored by calculating unweighted counts, percent distributions and then weighted percent distributions for each factor related to the child or parent/guardian. Weighted proportions of measles vaccination coverage were calculated among 2-year-old children and were stratified by sociodemographic characteristics of the child or parents/guardians.

The CNICS uses a complex sample design, and weighting is applied when deriving estimates to account for the unequal probability of participant selection by province/territory and age. A bootstrap resampling method was used in which 1000 bootstrap replicate weights were generated. Variance was estimated by calculating the value of the estimate of interest using each set of replicate weights and measuring the variability between these 1000 bootstrap estimates.

Next, to explore the association between the variables of interest and measles vaccination, univariate and exploratory multivariable logistic regression analyses were undertaken, and unadjusted and adjusted odds ratios were derived. Univariate logistic regression analyses were conducted to examine the relationship between up-to-date measles vaccination status and the selected predictor variables. Unadjusted odds ratios were presented to show the association between independent variables and up-to-date measles vaccination. Subsequently, an adjusted multivariate logistic regression model was applied to examine all correlates and their association with up-to-date measles vaccination.

A model fit was assessed by examining the Archer–Lemeshow goodness-of-fit test. Some predictor variables were excluded from the final model due to multicollinearity with other variables (i.e., remoteness index with urbanicity, visible minority with ethnicity) and the decision of which variable to include was determined based on best fit after fitting the model with each variable.

STATA version 17 for Windows was used for all analyses.

## 3. Results

### 3.1. Description of the Study Sample

Overall, 91.6% (95% CI: 89.9–93.1) of two-year-olds were up-to-date for measles vaccination. Regarding sample distribution, a higher proportion of 2-year-old children who were up-to-date for measles vaccination resided in Ontario (37.4%, 95% CI: 35.8–39.0), had parents/guardians who identified as a woman (87.8%, 95% CI: 85.7–89.6), had parents between the ages of 30–39 years (69.8%, 95% CI: 67.2–72.2), had parents with an education level of Bachelors’ degree or higher (54.0%, 95% CI: 51.2–17.5), had an average annual household income in 2020 of $100–149 K (30.8%, 95% CI: 28.5–33.2), were of White ethnicity (62.3%, 95% CI: 59.7–65.0), lived in an urban region (86.2%, 95% CI: 84.3–87.9) and lived in an easily accessible or accessible area (95.5%, 95% CI: 94.5–96.2) (Table 1).

In addition, the proportion of children who were up-to-date for measles vaccination by two years of age was higher among those with parents/guardians who indicated that they did not experience any delays or obstacles to vaccination that were due to the COVID-19 pandemic (86.3%, 95% CI: 84.3–88.1). The average vaccine hesitancy score was lower among parents/guardians of children who were up-to-date (Mean 1.40, 95% CI: 1.38–1.43) compared to those of unvaccinated children (Mean 2.03, 95% CI: 1.85–2.21) (Table 1).

### 3.2. Factors Associated with Up-to-Date Measles Vaccination

A total of 3118 two-year-old children were included in the final multivariable model. Several factors significantly associated with measles vaccination status in the univariate analyses were no longer statistically significant after adjustment, including parental age, education and marital status, region of residence, and child’s ethnicity. In contrast, lower household income, experiencing pandemic-related obstacles, and a higher hesitancy score remained independently associated with lower odds of being up-to-date with measles vaccination. These associations were statistically significant in both the univariate and multivariate analyses (Table 2).

Compared with children living in households with an annual income of $60,000–$99,999 in 2020, children from households with an annual income of less than $60,000 had 66% lower adjusted odds of being up-to-date with measles vaccination (aOR = 0.34, 95% CI: 0.16–0.71; *p* < 0.05). Similarly, children whose parents reported delaying or experiencing an obstacle to vaccination because of the COVID-19 pandemic had 77% lower adjusted odds of being up-to-date with measles vaccination compared to those who did not report an obstacle or delay (aOR = 0.23, 95% CI: 0.12–0.43, *p* < 0.05). In addition, each one-point increase in parental hesitancy score was associated with 51% lower adjusted odds of a 2-year-old child being up-to-date with measles vaccination (aOR = 0.49, 95% CI: 0.26–0.94, *p* < 0.05) (Table 2).

## 4. Discussion

This study identified several independent factors associated with up-to-date measles vaccination among two-year-old Canadian children. Lower household income, COVID-19 related vaccination delays or obstacles, and higher parental vaccine hesitancy remained significantly associated with measles vaccination after adjustment for other sociodemographic characteristics. These findings suggest that both structural barriers and parental attitudes influence routine childhood measles vaccination in Canada.

This study found that children from households with an annual income of less than $60,000 had significantly lower adjusted odds of being up-to-date with measles vaccination than children from households with annual incomes of $60,000–$99,999. This finding is consistent with previous studies that have reported an association between lower household income and lower measles vaccination coverage [17,23,24]. Although Canada’s routine childhood immunization programs are publicly funded, socioeconomic gaps in vaccine uptake exist. This ongoing income gradient reveals that equitable access is hindered by indirect financial costs and structural barriers rather than the cost of the vaccines themselves. Families with lower incomes may face indirect vaccination-related costs, such as limited access to healthcare facilities, limited flexibility to take time off work or transportation expenses for vaccination appointments [25]. Lower-income families may also experience reduced access to primary healthcare services or vaccine information, which can further hinder timely vaccination. In, addition, some studies suggest that parents with lower incomes may have greater concerns about vaccine safety and question the need for vaccination more frequently than those with higher incomes [25]. These findings suggest that improving measles vaccination coverage may require interventions that extend beyond providing vaccines at no cost, including improving access to vaccination services and reducing logistical barriers for lower-income families.

This analysis found that children whose parent or guardian reported delays or obstacles due to the COVID-19 pandemic had significantly lower odds of being up-to-date with measles vaccination than those whose parent or guardian did not report pandemic-related delays or obstacles. Public health restrictions, reduced healthcare capacity, and limited appointment availability created missed opportunities for routine childhood immunization, including measles. Consistent with these findings, most parents of two-year old children in the 2021 cNICS, identified “limited appointment availability” as the primary obstacle faced during the pandemic [5]. There is evidence that public health restrictions during the COVID-19 pandemic decreased access to vaccination programs and childhood vaccination coverage fell consequently in Canada [26,27]. Data from STARVAX has also reported a decline in one-dose MMR vaccination coverage among 2 yr olds following the onset of the COVID-19 pandemic, with coverage continuing to decline through 2025 [7]. Furthermore, in 2023, approximately one-quarter of Canadian parents reported missing or delaying a routine vaccine for their child since the beginning of the pandemic [28]. Other research suggests that children experience more delays in completing measles vaccination series due to obstacles related to caring for other siblings [29]. This is of particular concern as delays in vaccination can increase the risk of vaccine-preventable diseases and outbreaks.

Lastly, this study found that higher parental vaccine hesitancy was independently associated with lower odds of children being up-to-date with measles vaccination which is consistent with other research [29]. This finding suggests that parental attitudes toward vaccination continue to play an important role in childhood vaccine uptake, independent of sociodemographic factors. Despite the association between hesitancy and not being up-to-date on vaccination, most parents and guardians participating in the 2021 cNICS continued to report positive attitudes toward childhood vaccination, with over 97% agreeing that vaccines are safe, effective, and protect their child’s health [5]. Nevertheless, the COVID-19 pandemic was accompanied by increased vaccine misinformation and declining confidence in vaccination [30]. Consistent with this trend, a greater proportion of parents participating in the 2021 cNICS believed that alternative health practices (e.g., homeopathy or naturopathy) or healthy lifestyles could replace vaccination compared with 2019 [5]. These findings highlight the importance of maintaining public confidence in childhood vaccination through evidence-based communication and timely responses to vaccine misinformation.

Although this study did not examine parents’ sources of vaccine information, the literature suggests that the types of health information sources play an important role in shaping vaccine confidence and vaccination decisions [29]. In Canada, healthcare providers, particularly physicians, remain the most trusted sources of vaccine information among Canadian parents, followed by nurses and medical associations [5]. Those who seek information from social media and online sources may encounter vaccine misinformation stoking concerns surrounding confidence in vaccine safety and effectiveness [31,32]. It has also been shown that having access to a family doctor helps reduce the duration of vaccine delays [33]. Together, these findings suggest that strengthening communication between healthcare providers and parents, while addressing vaccine misinformation through trusted public health messaging, may improve vaccine confidence, supporting timely vaccination and fewer delays.

Though, ethnicity was not independently associated with measles vaccination in this analyses, other research suggests that differences in vaccination coverage could be explained, at least in part, by socioeconomic or other structural barriers rather than ethnicity itself. Canadian research has identified barriers to routine childhood vaccination among some racialized and Indigenous populations, including discrimination when accessing healthcare, language and communication challenges, reduced trust in healthcare institutions, lack of understanding of cultural values, and lack of accessibility [34,35]. Continued research is needed to better understand and address these barriers to ensure equitable access to childhood immunization.

In addition, targeted interventions should focus on under-vaccinated populations through culturally appropriate and accessible targeted public health strategies. Currently, through the Immunization Partnership Fund (IPF), the PHAC supports community-based initiatives aimed at increasing awareness of the importance of vaccination across the life course, strengthening vaccine confidence, and reducing barriers to accessing vaccination and vaccine education [36]. Expanding education and outreach through social platforms may also help reach diverse parent and guardian populations while countering vaccine misinformation.

### Strengths and Limitations

A limitation of this study is that approximately 35% of the immunization history among 2 yr olds in the 2021 cNICS relied on parent-held records which may be incomplete or erroneous. The survey excluded First Nations children living on-reserve, children in foster care, and institutionalized children. Furthermore, although results show a strong correlation between measles vaccination and obstacles experienced during the pandemic, the 2021 cNICS cycle may not have fully reflected the impact of the COVID-19 pandemic on 2-year-old children, since a portion of their routine vaccinations would have been received prior to the pandemic.

Raw numbers could not be provided. Statistics Canada is legally required under the Statistics Act to protect the confidentiality of information collected from individuals, households, businesses, and organizations. As a result, researchers who access Statistics Canada microdata through secure research environments may only publish results that have undergone a confidentiality review prior to release. To ensure that no individual person, household, business, or organization can be directly or indirectly identified from published findings, Statistics Canada applies statistical disclosure control measures before results can be released outside a secure research environment. Disclosure control measures include minimum count requirements, weighting, rounding, and minimum population counts for geographical areas. The purpose of these rules is to maintain the trust of Canadians and businesses, to fulfil the agency’s legal obligations, and to protect the confidentiality of respondents while also ensuring that valuable research can be conducted and disseminated.

Despite these limitations, this study has several strengths, including its use of nationally representative data and accounting for the complex survey design through the application of survey and bootstrap weights, improving the generalizability and precision of the findings. The multivariable analysis provided independent factors associated with measles vaccination, providing evidence to inform targeted public health interventions.

## 5. Conclusions

Maintaining high population immunity through routine childhood measles vaccination is essential for preventing outbreaks and supporting the restoration and maintenance of measles’s elimination in Canada. Furthermore, the WHO’s Immunization Agenda 2030 highlights the global importance of “measles as a tracer”, where measles cases, outbreaks, immunization, surveillance, and strategies can be indicators of the health and performance of entire immunization programs [37].

The findings from this analysis highlight several factors associated with measles vaccination among 2-year-olds from the 2021 cNICS. Lower household income, COVID-19-related delays or obstacles, and higher parental vaccine hesitancy were independently associated with measles vaccination. These findings emphasize the importance of considering both the social determinants of health and knowledge, attitudes, and beliefs around vaccination when developing and implementing public health immunization programs. Tailored interventions that address structural barriers to vaccination while strengthening vaccine confidence may help improve measles vaccination coverage, particularly among under-vaccinated populations. Continued surveillance of measles vaccination coverage in Canada remains essential for monitoring population immunity, identifying inequalities in vaccine uptake, and aiding public health strategies aimed at restoring and sustaining measles elimination.

## Figures and Tables

**Table 1:** Sample characteristics of 2-year-olds among children with known measles vaccination status, 2021 childhood National Immunization Coverage Survey.

Variable	Overall	Not Up-To-Date (0 Doses) Weighted % (95% CI) *	Up-To-Date (1 + Dose) Weighted % (95% CI) *
**Overall**—2 yr olds (N = 3118)		8.4 (6.9–10.1)	91.6 (89.9–93.1)
**Regions ^1^**			
Atlantic	5.1 (4.9–5.3)	3.1 (2.2–4.6)	5.3 (5.0–5.5)
Ontario	37.3 (36.0–38.6)	35.6 (25.8–46.8)	37.4 (35.8–39.0)
Quebec	25.9 (25.1–26.7)	21.0 (14.6–29.4)	26.3 (25.3–27.4)
Prairies	19.6 (18.8–20.4)	26.9 (20.5–34.4)	18.9 (18.0–19.9)
British Columbia	11.9 (11.3–12.5)	12.4 (8.1–18.5)	11.8 (11.1–12.6)
Territories	0.3 (0.3–0.3)	0.9 (0.7–1.2)	0.2 (0.2–0.3)
**Parent gender**			
Man	12.0 (10.2–13.9)	9.3 (5.2–16.3)	12.2 (10.4–14.3)
Woman	88.0 (86.1–89.8)	90.7 (83.7–94.9)	87.8 (85.7–89.6)
**Marital status**			
Married/common-law	88.9 (87.0–90.7)	72.5 (62.0–81.0)	90.4 (88.5–92.1)
Single	11.5 (9.3–13.0)	27.5 (19.0–11.5)	9.6 (7.9–11.5)
**Parent/guardian age**			
15–29 yrs	16.1 (14.3–18.1)	29.8 (21.2–40.0)	15.0 (13.1–17.0)
30–39 yrs	68.7 (66.2–71.1)	56.4 (46.2–66.1)	69.8 (67.2–72.2)
40–72 yrs	15.2 (13.4–17.2)	13.8 (8.3–22.1)	15.3 (13.4–17.4)
**Highest level of education of parent/guardian**			
High school or less	17.6 (15.6–19.8)	43.0 (33.4–53.1)	15.3 (13.4–17.5)
Above high school, below Bachelor’s (trades, colleges, certificate)	30.4 (27.9–32.9)	27.1 (19.0–37.1)	30.7 (28.1–33.3)
Bachelor’s or higher	52.1 (49.4–54.7)	29.9 (21.4–40.0)	54.0 (51.2–56.8)
**Household income—2020**			
Less than $60,000	17.4 (15.4–19.6)	41.9 (32.1–52.4)	15.2 (13.2–17.5)
$60,000–$99,999	26.8 (24.6–29.1)	27.9 (20.1–37.4)	26.7 (24.4–29.1)
$100,000–$149,999	29.8 (27.6–32.1)	18.8 (13.0–26.4)	30.8 (28.5–33.2)
$150,000 and above	26.0 (23.8–28.4)	11.4 (6.5–19.1)	27.4 (25.0–29.9)
**Child’s gender**			
Girl	49.3 (48.1–50.5)	46.9 (37.4–56.7)	49.5 (48.0–50.9)
Boy	50.7 (49.5–51.9)	53.1 (43.3–62.6)	50.5 (49.1–52.0)
**Child’s ethnicity**			
Indigenous	3.0 (2.3–4.0)	6.4 (3.1–12.9)	2.7 (2.0–3.7)
White	62.2 (59.7–64.7)	59.9 (49.7–69.3)	62.3 (59.7–65.0)
Asian/other	28.4 (26.1–30.8)	20.2 (13.7–28.8)	29.1 (26.7–31.7)
Black	6.4 (5.1–8.0)	13.5 (4.0–29.5)	5.7 (4.5–7.2)
**Urban rural**			
Rural	14.3 (12.6–16.2)	20.2 (13.5–29.1)	13.8 (12.1–15.7)
Urban	85.7 (83.8–87.4)	79.8 (70.9–86.5)	86.2 (84.3–87.9)
**Delays and obstacles due to COVID-19 pandemic**			
Yes	15.1 (13.2–17.2)	37.8 (26.5–50.7)	13.7 (11.9–15.7)
No	84.9 (82.8–86.8)	62.2 (49.4–73.6)	86.3 (84.3–88.1)
**Vaccine hesitancy score**			
Mean ± SD	1.46 (1.43–1.48)	2.03 (1.85–2.21)	1.40 (1.38–1.43)

Proportions may not add up to 100% due to rounding. * CI = confidence interval. ^1^ Atlantic region includes Nova Scotia, New Brunswick, Prince Edward Island, and Newfoundland and Labrador; Prairies include Alberta, Saskatchewan, and Manitoba; Territories include Northwest Territories, Nunavut, and Yukon.

**Table 2:** Univariate and adjusted odds ratios (weighted) from logistic regression analysis of the association between sociodemographic variables and up-to-date measles vaccination among 2-year-olds, 2021 childhood National Immunization Coverage Survey (cNICS).

Characteristic	Unadjusted OR (95% CI)	*p*-Value	Adjusted OR (95% CI)	*p*-Value
**Gender of Parent**				
Man	Reference		Reference	
Woman	0.74 [0.37–1.48]	0.39	0.44 [0.15–1.34]	0.15
**Parent/Guardian Age**				
15–29	0.41 [0.24–0.68]	**<0.001**	0.65 [0.30–1.40]	0.27
30–39	Reference		Reference	
40–72	0.89 [0.48–1.67]	0.72	0.86 [0.33–2.22]	0.76
**Education of Parent/Guardian**				
High school or less	Reference		Reference	
Trades/college/university certificate	3.17 [1.84–5.44]	**<0.001**	1.63 [0.76–3.51]	0.21
Bachelor’s or higher	5.06 [3.02–8.49]	**<0.001**	2.19 [0.97–4.91]	0.76
**Household Income (2020)**				
<$60,000	0.38 [0.22–0.66]	**<0.001**	0.34 [0.16–0.71]	**<0.001**
$60,000–$99,999	Reference		Reference	
$100,000–$149,999	1.71 [1.01–2.91]	0.05	0.93 [0.41–2.21]	0.87
$150,000+	2.52 [1.23–5.20]	0.01 *	0.92 [0.32–2.61]	0.87
**Marital Status**				
Single	0.28 [0.16–0.48]	**<0.001**	0.35 [0.27–1.59]	0.35
Married/common-law	Reference		Reference	
**Region ^1^**				
Atlantic	1.60 [0.89–2.88]	0.12	1.27 [0.54–3.01]	0.58
Quebec	1.19 [0.63–2.26]	0.59	1.07 [0.45–2.57]	0.86
Ontario	Reference		Reference	
Prairies	0.67 [0.39–1.15]	0.15	0.92 [0.32–1.93]	0.83
British Columbia	0.91 [0.46–1.78]	0.77	0.82 [0.32–2.09]	0.67
Territories	0.25 [0.14–0.43]	<0.001 *	0.49 [0.17–1.45]	0.20
**Child’s Gender**				
Boy	Reference		Reference	
Girl	1.11 (0.72–1.70)	0.64	1.41 90.79–2.52)	0.24
**Child’s Ethnicity**				
Indigenous	0.41 [0.17–0.99]	0.05	0.68 [0.16–2.85]	0.6
White	Reference		Reference	
Asian/other	1.38 [0.82–2.32]	0.22	1.23 [0.60–2.62]	0.54
Other	0.41 [0.19–0.89]	0.02 *	0.58 [0.21–1.65]	0.31
**Urban/Rural**				
Rural	0.63 [0.38–1.06]	0.08	1.15 [0.43–3.04]	0.79
Urban	Reference		Reference	
**Delays and Obstacles due to COVID-19 Pandemic**				
Yes	0.26 [0.15–0.46]	<0.001 *	0.23 [0.12–0.43]	<0.001 *
No	Reference		Reference	
**Knowledge, Attitudes, and Beliefs Alpha Score**				
**Vaccine Hesitancy Score**	0.15 [0.11–0.22]	<0.001 *	0.49 (0.26–0.94)	0.03 *

* <0.05. CI = confidence interval. ^1^ Atlantic region includes Nova Scotia, New Brunswick, Prince Edward Island, and Newfoundland and Labrador; Prairies include Alberta, Saskatchewan, and Manitoba; Territories include Northwest Territories, Nunavut, and Yukon.

## Data Availability

The authors do not have permission to share the data. Non-profit organizations, non-governmental organizations and the private sector can access Statistics Canada’s confidential microdata, depending on the eligibility of their project, either remotely in authorized workspaces via the VDL, onsite at the SDAC (formerly the FRDC) in Ottawa, or at a local RDC. Access fees vary depending on the project. https://www.statcan.gc.ca/en/microdata accessed between 1 October 2024 to 1 December 2025.

## Abbreviations

The following abbreviations are used in this manuscript:

aOR	Adjusted Odds Ratio
CCB	Canadian Child Tax Benefit
cNICS	Childhood National Immunization Coverage Survey
CI	Confidence Interval
IPF	Immunization Partnership Fund
KAB	Knowledge, Attitude, and Belief
MMR	Measles, Mumps, Rubella
MMRV	Measles, Mumps, Rubella, Varicella
OR	Odds Ratio
PHAC	Public Health Agency of Canada

## Appendix A

**Table A1:** Knowledge, attitude and belief statements asked to parents of two-year-olds included in the Vaccine Hesitancy Score, childhood National Immunization Coverage Survey (cNICS), 2021.

Statements	Response Options
In general, childhood vaccines are safe	1: Strongly agree 2: Somewhat agree 3: Somewhat disagree 4: Strongly disagree
2. In general, childhood vaccines are effective	
3. In general, vaccines help to protect my child’s health	
4. Please indicate to what extent you trust the following sources of information on immunization: **medical doctors**	
5. Please indicate to what extent you trust the following sources of information on immunization: **local Public Health Unit**	
6. Please indicate to what extent you trust the following sources of information on immunization: **Public Health Agency of Canada**	
